# Complete genome sequence of *Sebaldella termitidis* type strain (NCTC 11300^T^)

**DOI:** 10.4056/sigs.811799

**Published:** 2010-03-30

**Authors:** Miranda Harmon-Smith, Laura Celia, Olga Chertkov, Alla Lapidus, Alex Copeland, Tijana Glavina Del Rio, Matt Nolan, Susan Lucas, Hope Tice, Jan-Fang Cheng, Cliff Han, John C. Detter, David Bruce, Lynne Goodwin, Sam Pitluck, Amrita Pati, Konstantinos Liolios, Natalia Ivanova, Konstantinos Mavromatis, Natalia Mikhailova, Amy Chen, Krishna Palaniappan, Miriam Land, Loren Hauser, Yun-Juan Chang, Cynthia D. Jeffries, Thomas Brettin, Markus Göker, Brian Beck, James Bristow, Jonathan A. Eisen, Victor Markowitz, Philip Hugenholtz, Nikos C. Kyrpides, Hans-Peter Klenk, Feng Chen

**Affiliations:** 1 DOE Joint Genome Institute, Walnut Creek, California, USA; 2 ATCC- American Type Culture Collection, Manassas, Virginia, USA; 3 Los Alamos National Laboratory, Bioscience Division, Los Alamos, New Mexico, USA; 4 Biological Data Management and Technology Center, Lawrence Berkeley National Laboratory, Berkeley, California, USA; 5 Oak Ridge National Laboratory, Oak Ridge, Tennessee, USA; 6 DSMZ – German Collection of Microorganisms and Cell Cultures GmbH, Braunschweig,; 7 University of California Davis Genome Center, Davis, California, USA

**Keywords:** anaerobic, mesophile, nonmotile, non-sporeforming, Gram-negative, termite intestine, ‘*Fusobacteria*’, ‘*Leptotrichiaceae*’, GEBA

## Abstract

*Sebaldella termitidis* (Sebald 1962) Collins and Shah 1986, is the only species in the genus *Sebaldella* within the fusobacterial family ‘*Leptotrichiaceae*’*.* The sole and type strain of the species was first isolated about 50 years ago from intestinal content of Mediterranean termites. The species is of interest for its very isolated phylogenetic position within the phylum *Fusobacteria* in the tree of life, with no other species sharing more than 90% 16S rRNA sequence similarity. The 4,486,650 bp long genome with its 4,210 protein-coding and 54 RNA genes is part of the *** G****enomic* *** E****ncyclopedia of* *** B****acteria and* *** A****rchaea * project.

## Introduction

Strain NCTC 11300^T^ (= ATCC 33386^TM^ = NCTC 11300) is the type strain of the species *Sebaldella termitidis* [1]. The strain was first isolated from posterior intestinal content of *Reticulitermes lucifugus* (Mediterranean termites) by the French microbiologist Madeleine Sebald [1,2], and was initially classified as *Bacteroides termitidis* [3]. The unusually low G+C content, as well as biochemical features which did not correspond to those known for the other members of the genus *Bacteroides* [4], and the subsequently described novel 16S rRNA sequences [5] made the position of *B. termitidis* within the genus *Bacteroides* appear controversial, and guided Collins and Shah in 1986 to reclassify *B. termitidis* as the type strain of the novel genus *Sebaldella* [1]. Here we present a summary classification and a set of features for *S. termitidis* NCTC 11300^T^, together with the description of the complete genomic sequencing and annotation.

## Classification and features

NCTC 11300^T^ represents an isolated species, with no other cultivated strain known in the literature belonging to the species. An uncultured clone with identical 16S rRNA sequence was identified in a mesophilic anaerobic digester that treats municipal wastewater sludge in Clos de Hilde, France [6], and another uncultured clone, PCD-1 (96.1% 16S rRNA sequence identity), was reported from the digestive tract of the ground beetle *Poecilus chalcites* [7]. The closest related type strains are those of the genus *Leptotrichia*, which share 85.9 to 89.96% 16S rRNA sequence similarity [8]. Neither environmental screenings nor metagenomic surveys provided any 16S rRNA sequence with significant sequence similarity to NCTC 11300^T^, indicating that members of the species *S. termitidis* and the genus *Sebaldella* are not very frequent in the environment (status February 2010).

**Figure 1:**
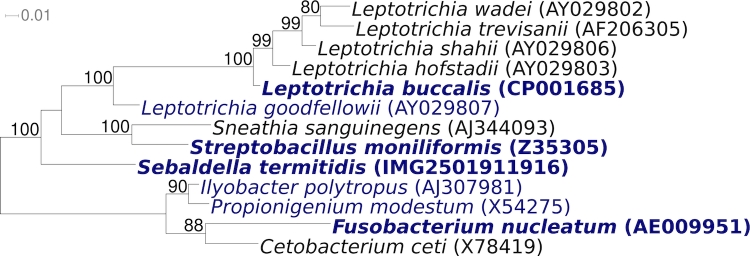
Phylogenetic tree highlighting the position of *S. termitidis* NCTC 11300^T^ relative to the other type strains within the family ‘*Leptotrichiaceae*’. The tree was inferred from 1,422 aligned characters [9,10] of the 16S rRNA gene sequence under the maximum likelihood criterion [11] and rooted in accordance with the current taxonomy. The branches are scaled in terms of the expected number of substitutions per site. Numbers above branches are support values from 1,000 bootstrap replicates if larger than 60%. Lineages with type strain genome sequencing projects registered in GOLD [12] are shown in blue, published genomes in bold, e.g. the recently published GEBA genomes from *Leptotrichia buccalis* [13], and *Streptobacillus moniliformis* [14].

Figure 1 shows the phylogenetic neighborhood of *S. termitidis* NCTC 11300^T^ in a 16S rRNA based tree. The sequences of the four identical copies of the 16S rRNA gene in the genome do not differ from the previously published 16S rRNA sequence generated from ATCC 3386 (M58678), which is missing two nucleotides and contains 30 ambiguous base calls.

**Figure 2:**
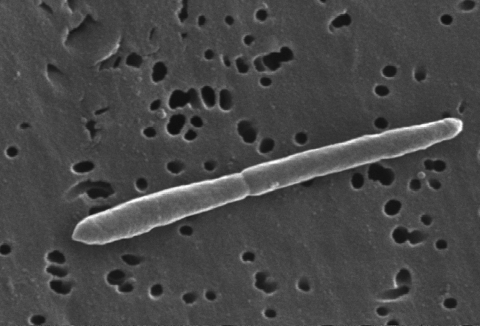
Scanning electron micrograph of *S. termitidis* NCTC 11300^T^. (J. Carr, CDC, Atlanta, Georgia). More EM photos of the organism can be found at http://phil.cdc.gov/phil.

Cells of strain NCTC 11300^T^ are Gram-negative, obligately anaerobic, nonmotile, nonspore-forming rods of 0.3 to 0.5 x 2 to 12 μm with central swellings (Figure 2 and Table 1) [1]. Cells occur singly, in pairs, as well as in filaments [1]. Colonies on surface are transparent to opaque, circular measuring 1-2 mm in diameter, whereas colonies in deep agar are non pigmented and lenticular [1].

**Table 1:** Classification and general features of *S. termitidis* NCTC 11300^T^ according to the MIGS recommendations [15]

**MIGS ID**	**Property**	**Term**	**Evidence code**
	Current classification	Domain *Bacteria*	TAS [16]
		Phylum *Fusobacteria*	TAS [17]
		Class ‘*Fusobacteria*’	TAS [17]
		Order ‘*Fusobacteriales*’	TAS [17]
		Family ‘*Leptotrichiaceae*’	TAS [18]
		Genus *Sebaldella*	TAS [1,19]
		Species *Sebaldella termitidis*	TAS [1,19]
		Type strain NCTC 11300	TAS [1]
	Gram stain	Gram negative	TAS [1]
	Cell shape	rod-shaped, with central swellings; occur singly, in pairs and in filaments	TAS [1]
	Motility	nonmotile	TAS [1]
	Sporulation	nonsporulating	TAS [2]
	Temperature range	mesophile	NAS
	Optimum temperature	not determined	
	Salinity	not reported	
MIGS-22	Oxygen requirement	obligate anaerobic	TAS [1]
	Carbon source	glucose and other sugars	TAS [1]
	Energy source	fermentation of glucose and other sugars	TAS [1]
MIGS-6	Habitat	bacterial flora of termite gastrointestinal tract	TAS [1]
MIGS-15	Biotic relationship	unknown	
MIGS-14	Pathogenicity	none reported	NAS
	Biosafety level	2	TAS [20]
	Isolation	posterior intestinal content of termites	TAS [2]
MIGS-4	Geographic location	unknown	
MIGS-5	Sample collection time	1962 or before	TAS [1,2]
MIGS-4.1 MIGS-4.2	Latitude Longitude	not reported	
MIGS-4.3	Depth	not reported	
MIGS-4.4	Altitude	not reported	

Evidence codes - IDA: Inferred from Direct Assay (first time in publication); TAS: Traceable Author Statement (i.e., a direct report exists in the literature); NAS: Non-traceable Author Statement (i.e., not directly observed for the living, isolated sample, but based on a generally accepted property for the species, or anecdotal evidence). These evidence codes are from of the Gene Ontology project [21]. If the evidence code is IDA, then the property was directly observed for a live isolate by one of the authors or an expert mentioned in the acknowledgements.

The major end products of the glucose metabolism by strain NCTC 11300^T^ are acetic and lactic acids (with some formic acid) as opposed to succinic and acetic acids dominating in members of the genus *Bacteroides* [1]. Enzymes of the hexose-monophosphate-shunt are missing, while present in members of the genus *Bacteroides* [1,4]. A list of additional sugars and alcohols used or not-used for fermentation is provided by Collins and Shah [1].

### Chemotaxonomy

The cell wall structure of strain NCTC 11300^T^ has not yet been reported. Nonhydroxylated and 3-hydroxyated fatty acids were present [1]. The major long chain fatty acids are saturated and monounsaturated straight chain acids: C_16:0_ (37%) and C_18:1_ (41%), with methyl branched acids being absent [1], as opposed to straight-chain saturated, anteiso- and iso-methyl branched-chain acids in members of the genus *Bacteroides*, which are missing the monounsaturated acids [1]. Menaquinones were not detected, as opposed to members of the genus *Bacteroide*s [1].

## Genome sequencing and annotation

### Genome project history

This organism was selected for sequencing on the basis of its phylogenetic position, and is part of the *** G****enomic* *** E****ncyclopedia of* *** B****acteria and* *** A****rchaea * project [22]. The genome project is deposited in the Genome OnLine Database [12] and the complete genome sequence is deposited in GenBank. Sequencing, finishing and annotation were performed by the DOE Joint Genome Institute (JGI). A summary of the project information is shown in Table 2.

**Table 2:** Genome sequencing project information

**MIGS ID**	**Property**	**Term**
MIGS-31	Finishing quality	Finished
MIGS-28	Libraries used	One genomic 8kb pMCL200 library, one 454 pyrosequence library and one Illumina library
MIGS-29	Sequencing platforms	Sanger, 454 Titanium, Illumina
MIGS-31.2	Sequencing coverage	9.2× Sanger; 30.3× 454 Titanium
MIGS-30	Assemblers	Newbler, phrap
MIGS-32	Gene calling method	Prodigal, GenePRIMP
	INSDC ID	CP001739 (chromosome), CP001740, CP001741 (plasmids)
	Genbank Date of Release	November 19, 2009
	GOLD ID	Gc01144
	NCBI project ID	29539
	Database: IMG-GEBA	2501846314
MIGS-13	Source material identifier	ATCC 33386
	Project relevance	Tree of Life, GEBA

### Growth conditions and DNA isolation

*S. termitidis* NCTC 11300^T^, ATCC 33386^TM^, was grown anaerobically in ATCC medium 1490 (Modified chopped meat medium) [23] at 37°C. DNA was isolated from cell paste using a basic CTAB extraction and then quality controlled according to JGI guidelines.

### Genome sequencing and assembly

The genome was sequenced using a combination of Sanger and 454 sequencing platforms. All general aspects of library construction and sequencing can be found at http://www.jgi.doe.gov/. 454 Pyrosequencing reads were assembled using the Newbler assembler version 1.1.02.15 (Roche). Large Newbler contigs were broken into 4,966 overlapping fragments of 1,000 bp and entered into assembly as pseudo-reads. The sequences were assigned quality scores based on Newbler consensus q-scores with modifications to account for overlap redundancy and to adjust inflated q-scores. A hybrid 454/Sanger assembly was made using the parallel phrap assembler (High Performance Software, LLC). Possible mis-assemblies were corrected with Dupfinisher [24] or transposon bombing of bridging clones (Epicentre Biotechnologies, Madison, WI). Gaps between contigs were closed by editing in Consed, custom primer walk or PCR amplification. A total of 796 Sanger finishing reads were produced to close gaps, to resolve repetitive regions, and to raise the quality of the finished sequence. Illumina reads were used to improve the final consensus quality using an in-house developed tool (the Polisher, unpublished). The error rate of the completed genome sequence is less than 1 in 100,000. Together all sequence types provided 39.5× coverage of the genome. The final assembly contains 45,934 Sanger and 760,187 pyrosequence reads.

### Genome annotation

Genes were identified using Prodigal [25] as part of the Oak Ridge National Laboratory genome annotation pipeline, followed by a round of manual curation using the JGI GenePRIMP pipeline [26]. The predicted CDSs were translated and used to search the National Center for Biotechnology Information (NCBI) nonredundant database, UniProt, TIGRFam, Pfam, PRIAM, KEGG, COG, and InterPro databases. Additional gene prediction analysis and manual functional annotation was performed within the Integrated Microbial Genomes Expert Review (IMG-ER) platform [27].

## Genome properties

**Figure 3:**
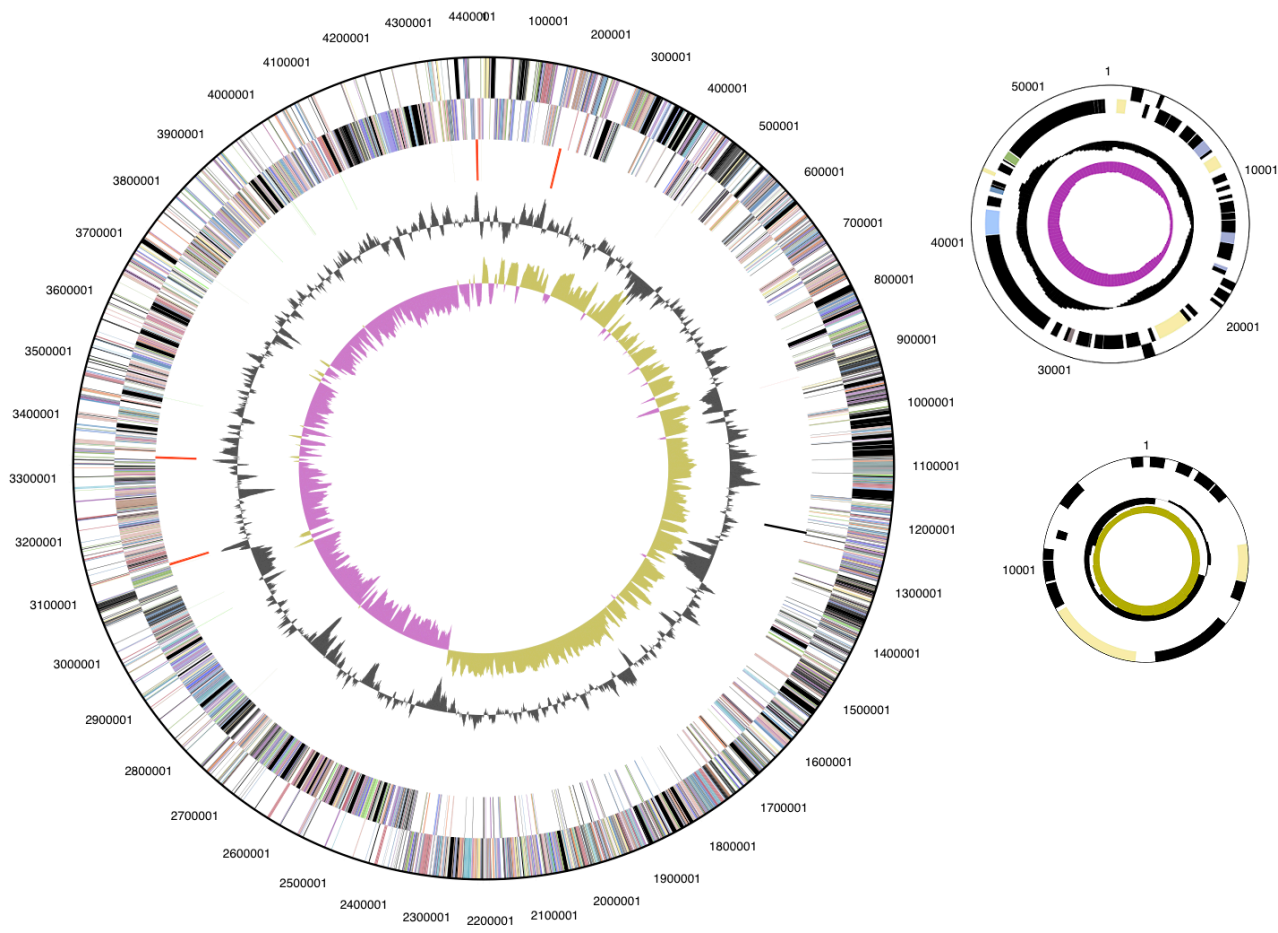
Graphical circular maps of the chromosome and the two plasmids. From outside to the center: Genes on forward strand (color by COG categories), Genes on reverse strand (color by COG categories), RNA genes (tRNAs green, rRNAs red, other RNAs black), GC content, GC skew.

The genome consists of a 4,418,842 bp long chromosome, and two plasmids with 54,160 bp and 13,648 bp length, respectively, with a 33.4% GC content (Table 3 and Figure 3). Of the 4,264 genes predicted, 4,210 were protein-coding genes, and 54 RNAs; 59 pseudogenes were identified. The majority of the protein-coding genes (60.4%) were assigned with a putative function while those remaining were annotated as hypothetical proteins. The distribution of genes into COGs functional categories is presented in Table 4.

**Table 3:** Genome Statistics

**Attribute**	**Value**	**% of Total**
Genome size (bp)	4,486,650	100.00%
DNA coding region (bp)	3,918,335	87.33%
DNA G+C content (bp)	1,497,450	33.38%
Number of replicons	3	
Extrachromosomal elements	2	
Total genes	4,264	100.00%
RNA genes	54	1.27%
rRNA operons	4	
Protein-coding genes	4,210	98.73%
Pseudogenes	59	1.38%
Genes with function prediction	2,576	60.41%
Genes in paralog clusters	1,253	29.39%
Genes assigned to COGs	2,299	60.95%
Genes assigned Pfam domains	2,787	65.36%
Genes with signal peptides	801	18.79%
Genes with transmembrane helices	901	21.13%
CRISPR repeats	1	

**Table 4:** Number of genes associated with the general COG functional categories

**Code**	**value**	**%age**	**Description**
J	152	3.6	Translation, ribosomal structure and biogenesis
A	0	0.0	RNA processing and modification
K	265	6.3	Transcription
L	130	3.1	Replication, recombination and repair
B	0	0.0	Chromatin structure and dynamics
D	22	0.5	Cell cycle control, cell division, chromosome partitioning
Y	0	0.0	Nuclear structure
V	47	1.1	Defense mechanisms
T	96	2.3	Signal transduction mechanisms
M	155	3.7	Cell wall/membrane biogenesis
N	17	0.4	Cell motility
Z	0	0.0	Cytoskeleton
W	0	0.0	Extracellular structures
U	41	1.0	Intracellular trafficking, secretion and vesicular transport
O	71	1.7	Posttranslational modification, protein turnover, chaperones
C	128	3.0	Energy production and conversion
G	468	11.1	Carbohydrate transport and metabolism
E	219	5.2	Amino acid transport and metabolism
F	93	2.2	Nucleotide transport and metabolism
H	106	2.5	Coenzyme transport and metabolism
I	59	1.4	Lipid transport and metabolism
P	105	2.5	Inorganic ion transport and metabolism
Q	32	0.8	Secondary metabolites biosynthesis, transport and catabolism
R	403	9.6	General function prediction only
S	241	5.7	Function unknown
-	1,665	39.5	Not in COGs
